# Integrated Transcriptomic and Metabolomic Analyses Reveal the Regulatory Drivers of Anthocyanin-Mediated Leaf Color Variation in *Liquidambar formosana*

**DOI:** 10.3390/ijms27125429

**Published:** 2026-06-16

**Authors:** Fangwei Zhou, Longjie Ni, Liang Xu, Congguang Shi, Shaozong Yang

**Affiliations:** Zhejiang Key Laboratory of Forest Genetics and Breeding, Zhejiang Academy of Forestry, Hangzhou 310023, China; zhoufangwei@njfu.edu.cn (F.Z.); longjieni@njfu.edu.cn (L.N.); xuliang@zjforestry.ac.cn (L.X.); shicongguang@zjforestry.ac.cn (C.S.)

**Keywords:** *Liquidambar formosana*, leaf color, anthocyanin, flavonoid

## Abstract

Seasonal changes in leaf coloration are a key ecological and ornamental characteristic of *Liquidambar formosana*. To clarify the molecular basis of this process, we performed an integrated transcriptomic and metabolomic investigation comparing wild-type *L. formosana* (FX) with the autumn-red cultivar ‘Jinyu’ (JY). Leaves were sampled before and after the color transition. Analyses revealed distinct metabolic pathways driving coloration in each genotype. In JY, the red phenotype was primarily attributed to the activation of anthocyanin biosynthesis, characterized by coordinated upregulation of key structural genes (*LfDFR*, *LfANS*, *LfBZ1*) and significant accumulation of anthocyanins, especially pelargonidin derivatives. Conversely, FX exhibited enhanced flavonol biosynthesis and carotenoid/terpenoid metabolism, leading to greater yellow pigment accumulation and an orange-yellow hue. Weighted gene co-expression network analysis (WGCNA) identified a core module strongly correlated with anthocyanin content in JY, which was significantly enriched with transcription factors from the MYB, bHLH, and WRKY families. These results demonstrate that different *L. formosana* genotypes employ divergent metabolic strategies for autumn coloration, governed by specific transcriptional regulatory networks. This study provides crucial insights into pigment regulation in woody plants and offers valuable candidate genes for the molecular breeding of ornamental *L. formosana* cultivars.

## 1. Introduction

Seasonal leaf color transformation is among the most striking visual phenomena in temperate ecosystems during autumn, and the molecular mechanisms governing this process have attracted sustained interest in plant biology. Leaf pigmentation is primarily determined by the relative abundance and composition of several classes of pigments, which include carotenoids, chlorophylls, and anthocyanins. Of these compounds, anthocyanin accumulation is widely recognized as the principal contributor to the red hues observed in autumn foliage [1]. In temperate climates, progressive shortening of photoperiods coupled with declining temperatures accelerates chlorophyll breakdown while simultaneously stimulating anthocyanin biosynthesis. These coordinated changes drive the visible shift in leaf coloration toward yellow, orange, or red tones [2]. Environmental cues such as temperature, light intensity, and sugar signaling modulate anthocyanin levels by influencing genes encoding biosynthetic enzymes. In particular, exposure to low temperatures and high irradiance generally enhances anthocyanin production, which in turn confers partial photoprotection by shielding the photosynthetic machinery from photooxidative stress [3].

Anthocyanin formation proceeds through a multistep enzymatic pathway involving numerous structural genes within the phenylpropanoid and flavonoid metabolic networks. The pathway is initiated with phenylalanine, which is sequentially processed by early enzymes such as phenylalanine ammonia-lyase (PAL) and 4-coumarate-CoA ligase (4CL). Subsequent reactions enter the core flavonoid biosynthetic steps catalyzed by chalcone synthase (CHS), chalcone isomerase (CHI), and flavanone 3-hydroxylase (F3H). The pathway culminates in anthocyanin synthesis through the activity of late, pathway-specific enzymes, including dihydroflavonol 4-reductase (DFR) and anthocyanidin synthase (ANS) [4,5,6]. A critical regulatory point within this network lies in the competition between flavonol synthase (FLS) and DFR for the shared substrate dihydroflavonols. While FLS diverts these intermediates toward flavonol production, DFR channels them into anthocyanin biosynthesis. The balance of carbon flux between these two enzymes is therefore considered a major determinant of overall anthocyanin accumulation efficiency [7,8].

Beyond this overall enzymatic framework, anthocyanin biosynthesis is tightly governed transcriptionally by an intricate network of regulatory factors. Extensive research has demonstrated that this biosynthetic pathway is evolutionarily conserved across plant species and can be broadly divided into a general phenylpropanoid pathway and an anthocyanin-specific branch [9,10]. Among the structural genes, PAL functions as a key entry-point enzyme by catalyzing the deamination of phenylalanine to trans-cinnamic acid, followed by coordinated expression of downstream genes like CHS, CHI, F3H, DFR, and ANS [11,12]. Variability in the expression patterns of these genes has been identified as a major contributor to genotypic differences in anthocyanin accumulation, largely driven by dynamic interactions within transcriptional regulatory networks. Central to this regulation is the MYB-bHLH-WD40 (MBW) complex, which is widely regarded as the core regulatory module controlling anthocyanin biosynthesis [13]. In addition, the identification of other transcription factor families, including NAC, WRKY, and bZIP proteins, highlights the existence of multilayered and context-dependent regulatory circuits that contribute to cultivar-specific patterns of leaf coloration.

*Liquidambar formosana* is a widely planted tree species in China, held in high esteem for afforestation, ornamental landscaping, traditional medicine, and resin production. Importantly, its leaves are naturally rich in anthocyanins and flavonoids, with 148 flavonoid metabolites (including 15 anthocyanins) having been recently characterized [14]. The anthocyanidin content peaks in autumn, directly contributing to the vivid red coloration of the foliage. These features make *L. formosana* a promising natural source of anthocyanins and a suitable model for investigating pigment metabolism in woody plants. Furthermore, the recent publication of a high-quality, chromosome-level genome assembly has revealed its basal phylogenetic position in core eudicots and a highly conserved genome structure [15], establishing *L. formosana* as an ideal reference for comparative genomic studies of secondary metabolism in woody perennials. Substantial variation in leaf color among cultivars or genetic lines offers a natural system for exploring the genetic and molecular determinants of anthocyanin accumulation. For instance, studies in purple-leaf tea (*Camellia sinensis*) have demonstrated that transcriptional control of anthocyanin biosynthetic genes is a primary driver of leaf pigmentation [16]. Similarly, in red-leaf cultivars of Chinese tallow (*Triadica sebifera*), the MYB transcription factor *SsMYB1* has been shown to promote red leaf formation by activating anthocyanin-related structural genes [17]. Comparable phenotypic diversity is observed in *L. formosana*: while typical wild-type individuals develop orange-yellow leaves in autumn, cultivars such as ‘Jinyu’ consistently display bright red foliage. This stable and pronounced contrast in leaf coloration provides an ideal foundation for comparative transcriptomic and metabolomic analyses.

Although previous studies have elucidated key aspects of anthocyanin biosynthesis, particularly with respect to the roles of structural genes and individual transcription factors, the broader regulatory network governing leaf color transitions in *L. formosana* remains incompletely characterized. Specifically, the coordinated regulatory functions of different transcription factor families, including MYB and WRKY proteins, and the interactions between the anthocyanin pathway and other metabolic processes have not been fully resolved. Moreover, integrative strategies that combine transcriptomic and metabolomic datasets have rarely been employed for studying leaf coloration in *L. formosana*, limiting a comprehensive understanding of its molecular regulation. To address these gaps, the present study used common *L. formosana* and the ‘Jinyu’ cultivar as experimental materials and employed transcriptomic, metabolomic, and systems biology approaches, including WGCNA, to systematically dissect the metabolic and transcriptional networks underlying their contrasting leaf color phenotypes. Importantly, beyond reporting the first multi-omics analysis of leaf coloration in this species, our study reveals that two genotypes of *L. formosana* (wild-type FX and the red-leaf cultivar ‘Jinyu’) employ fundamentally divergent metabolic strategies–a novel intra-specific comparison that uncovers previously unrecognized regulatory hubs, including WRKY and NAC transcription factors, within a co-expression network strongly correlated with anthocyanin accumulation. These findings provide not only a deeper understanding of pigment regulation in woody perennials but also provide a theoretical basis for the molecular improvement and targeted regulation of leaf color traits in woody ornamental species.

## 2. Results

### 2.1. Phenotypic and Physiological Changes in Leaves of ‘Jinyu’ and Wild-Type L. formosana

**Figure 1:**
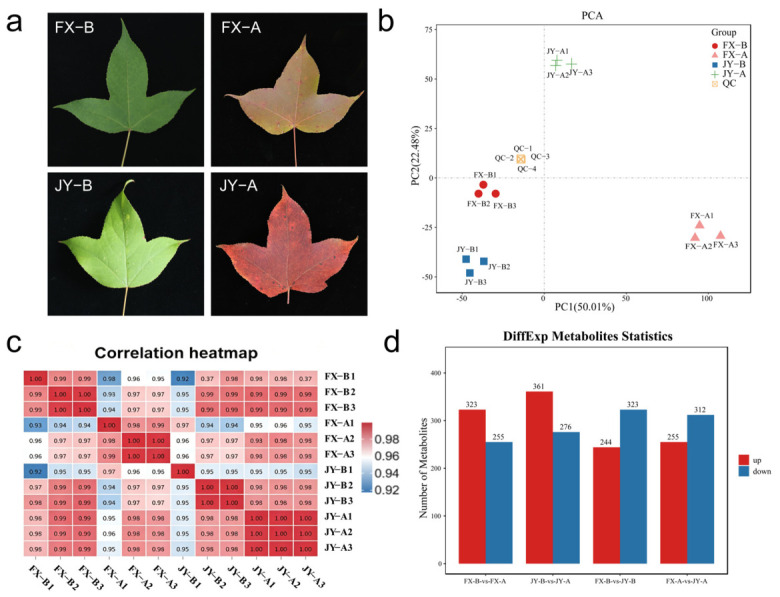
LC-MS/MS-based untargeted metabolomic profiling of leaf color transitions in wild-type *L. formosana* (FX) and *L. formosana* ‘Jinyu’ (JY). (**a**) Representative leaf phenotypes of FX and JY before color transition in summer (FX-B and JY-B) and after autumn coloration (FX-A and JY-A). FX-A shows orange-yellow leaves, while JY-A shows bright red leaves. (**b**) PCA score plot based on metabolite profiles. QC represents quality control samples (pooled aliquots of all samples) used to assess instrument stability. (**c**) Pearson correlation heatmap illustrating pairwise correlations among samples. (**d**) Numbers of differentially accumulated metabolites (DAMs) identified in each comparison.

To explore the metabolic mechanisms underlying divergent leaf coloration between wild-type *L. formosana* (FX) and the ‘Jinyu’ cultivar (JY) (Figure 1a), untargeted metabolomic profiling based on LC-MS/MS was performed on leaf samples collected before and after the color transition. The two genotypes exhibited distinct metabolic adjustment patterns during the coloration process. Principal component analysis (PCA) revealed that the first two principal components explained 50.01% (PC1) and 22.58% (PC2) of the total variance, respectively (Figure 1b). Clear separation among the four sample groups (FX-B, FX-A, JY-B, and JY-A) was observed in the PCA score plot. Notably, FX samples before and after coloration were primarily separated along PC1, whereas JY samples were mainly distinguished along PC2, indicating that the two genotypes relied on partially different metabolic pathways during leaf color development.

The correlation heatmap (Figure 1c) showed strong correlations among biological replicates within the same genotype and developmental stage (r > 0.98), confirming high data reproducibility. In contrast, correlations between different sample groups were markedly lower, suggesting that metabolic divergence between FX and JY was already present prior to visible color change. OPLS-DA further strengthened group discrimination, and model validation parameters confirmed the stability and reliability of the analytical models. Using variable importance in projection (VIP) values > 1.0 combined with FDR < 0.05 as selection criteria, 578 differentially accumulated metabolites were identified during the coloration process of wild-type FX (FX-B vs. FX-A), including 323 upregulated and 255 downregulated metabolites (Figure 1d). The statistical validation for key metabolites is provided in Table S1. In JY (JY-B vs. JY-A), 637 differential metabolites were observed, including 361 that were upregulated and 276 that were downregulated, indicating a broader scope of metabolic reprogramming during coloration in the red-leaf cultivar. Cross-genotype comparisons identified 567 differential metabolites before coloration (FX-B vs. JY-B), with 244 metabolites upregulated and 323 downregulated in FX relative to JY. A comparable number of differential metabolites (567) was observed after coloration (FX-A vs. JY-A), including 255 upregulated and 312 downregulated metabolites.

### 2.2. Metabolomic Profiling Highlights Divergence in Pigment-Associated Metabolic Pathways

**Figure 2:**
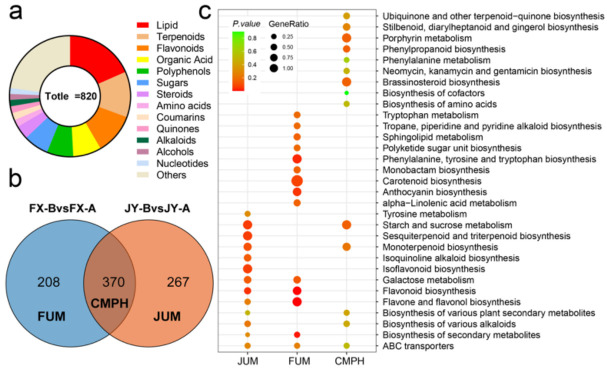
Pathway enrichment analyses for differential metabolites identified during leaf color transition in FX and JY. (**a**) Category distribution of the 820 differential metabolites identified from four pairwise comparisons (FX-B vs. FX-A, JY-B vs. JY-A, FX-B vs. JY-B, and FX-A vs. JY-A). (**b**) Venn diagram illustrating shared and cultivar-specific differential metabolites. FUM denotes metabolites specific to FX (FX-B vs. FX-A), JUM denotes metabolites specific to JY (JY-B vs. JY-A), and CMPH represents the 370 metabolites common to both cultivars. (**c**) KEGG pathway enrichment bubble plot for FUM, JUM, and CMPH metabolite groups.

Differential metabolite analyses demonstrated that wild-type FX and the JY cultivar underwent markedly different metabolic reprogramming during autumn leaf coloration. Across all pairwise comparisons, a total of 820 metabolites showed significant differential accumulation (Figure 2a). These compounds were predominantly assigned to lipid, flavonoid, terpenoid, organic acid, and polyphenol classes, collectively forming the biochemical basis of leaf color transformation in *L. formosana*. The distribution of these metabolites underscores the central involvement of phenylpropanoid-flavonoid and terpenoid pathways in regulating autumn pigmentation. Venn diagram analysis (Figure 2b) revealed that FX and JY shared 370 differential metabolites (CMPH), representing 45.1% of the total identified set. In contrast, 208 metabolites were uniquely altered in FX (FUM), whereas 267 were specific to JY (JUM). These findings indicate that, although a conserved metabolic framework supports leaf coloration in both genotypes, substantial cultivar-dependent metabolic divergence exists. Subsequent KEGG pathway enrichment analysis further emphasized these differences. FX-specific metabolites (FUM) were significantly enriched for pathways linked to anthocyanin biosynthesis, flavone and flavonol biosynthesis, and phenylalanine metabolism, suggesting that activation of anthocyanin-related pathways plays a dominant role in shaping the pigmentation of FX leaves. Conversely, JY-specific metabolites (JUM) were predominantly associated with carotenoid biosynthesis and sesquiterpenoid and monoterpenoid metabolism, consistent with the characteristic orange-yellow tones observed in this cultivar.

Consistent with these metabolic patterns, JY leaves developed a vivid red coloration, whereas FX leaves transitioned to a golden-orange hue during autumn (Figure 2a). Despite the large number of shared differential metabolites, the presence of numerous cultivar-specific compounds highlights that leaf coloration arises from a shared metabolic backbone that is modulated by genotype-specific pathway preferences. The KEGG enrichment heatmap (Figure 2c) clearly illustrates pronounced activation of anthocyanin and flavonoid biosynthesis in FX (FUM), while JY (JUM) exhibits stronger enrichment in carotenoid and terpenoid pathways. These pathway-level differences provide a mechanistic explanation for the contrasting red versus orange-yellow leaf phenotypes. In contrast, the shared metabolite set (CMPH) was mainly enriched in fundamental metabolic processes, including cofactor biosynthesis, amino acid metabolism, general secondary metabolite biosynthesis, multiple alkaloid biosynthetic pathways, and ABC transporter activity. These pathways likely constitute a core metabolic network that supports leaf color change in both cultivars.

### 2.3. Anthocyanin Metabolite Profiling

**Figure 3:**
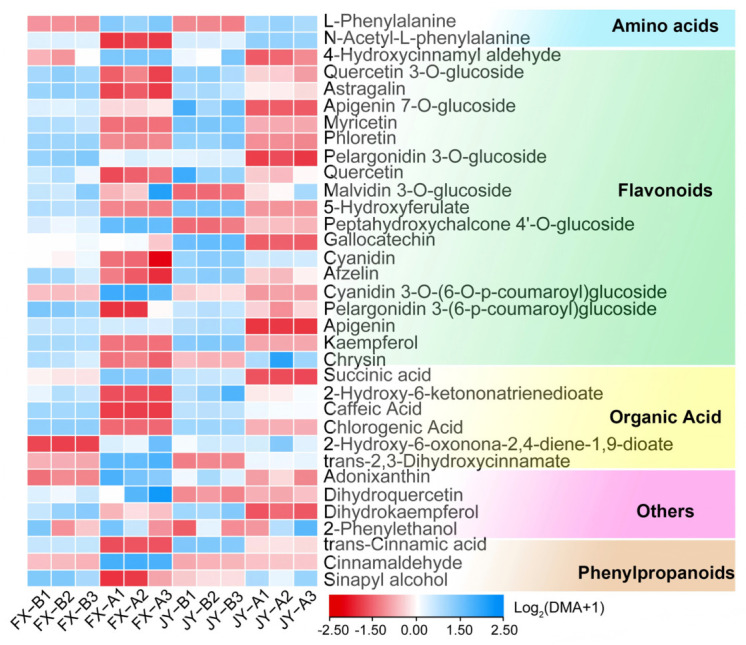
Heatmap illustrating the relative abundance of representative differential metabolites associated with the phenylpropanoid-flavonoid pathway during leaf coloration in FX and JY. Metabolite levels were normalized and visualized using a color gradient from blue to red, respectively denoting low and high abundance levels. The y-axis lists selected metabolites grouped by compound class (amino acids, flavonoids, organic acids, phenylpropanoids, and others), while the x-axis represents three biological replicates for each treatment (FX-B1-3, FX-A1-3, JY-B1-3, and JY-A1-3).

Targeted analysis of pigment-associated metabolites revealed substantial differences in anthocyanin accumulation between the wild-type FX cultivar, which develops orange-yellow foliage, and the red-leaf JY cultivar (Figure 3). In FX, before color transition in summer (FX-B) and after autumn coloration (FX-A), the stages showed pronounced shifts in anthocyanin composition. Prior to color change, concentrations of major anthocyanins, particularly cyanidin-derived compounds, were relatively low. Following coloration, however, levels of key anthocyanins, including cyanidin and malvidin 3-O-glucoside, along with their glycosylated and acylated derivatives, increased substantially. Notably, cyanidin accumulated to a high level in FX-A samples, while cyanidin 3-O-(6-O-p-coumaroyl)glucoside remained low. This suggests that free cyanidin, rather than its acylated derivative, may contribute to the orange-yellow pigmentation of FX leaves, which is less intense than the red coloration of JY. In parallel, several phenolic compounds, such as quercetin, quercetin 3-O-glucoside, and apigenin 7-O-glucoside, were significantly upregulated in FX after color transition. Upstream intermediates of the phenylpropanoid pathway, including N-acetyl-L-phenylalanine and 4-hydroxycinnamyl aldehyde, also showed increased abundance, indicating coordinated activation of the phenylpropanoid-flavonoid-anthocyanin metabolic cascade during the orange-yellow coloration of FX leaves. In contrast, anthocyanin metabolism in JY followed a distinct trajectory. After leaf color transition, JY-A samples showed strong accumulation of specific anthocyanins, such as pelargonidin 3-(6-p-coumaroyl)glucoside and pelargonidin 3-O-glucoside, as well as certain flavonoids including apigenin and kaempferol. In comparison, typical flavonols like quercetin and myricetin exhibited relatively modest changes in abundance.

Direct inter-cultivar comparisons further highlighted these differences. During the coloration of FX-A leaves, anthocyanins and related phenolic compounds increased in a broad and coordinated manner, encompassing a wide range of metabolites with large fold changes, particularly cyanidin and its derivatives. In JY-A leaves, however, pigmentation was driven by the pronounced accumulation of a narrower subset of anthocyanin subclasses and distinctive flavonoids, such as chrysin. These contrasting accumulation patterns underscore the strong influence of genetic background on the metabolic basis of autumn leaf coloration in *L. formosana*. Specifically, FX-A achieves its leaf color transition primarily through widespread activation of phenylpropanoid and anthocyanin biosynthesis, leading to generalized enhancement of anthocyanins and phenolics. In contrast, JY-A relies on selective enrichment of specific anthocyanin species and flavonoids to produce its characteristic red foliage. Together, these divergent metabolic strategies constitute the principal biochemical foundation underlying leaf color variation between the two cultivars.

### 2.4. Transcriptomic Profiling Reveals Distinct Molecular Bases of Leaf Color Differentiation

**Figure 4:**
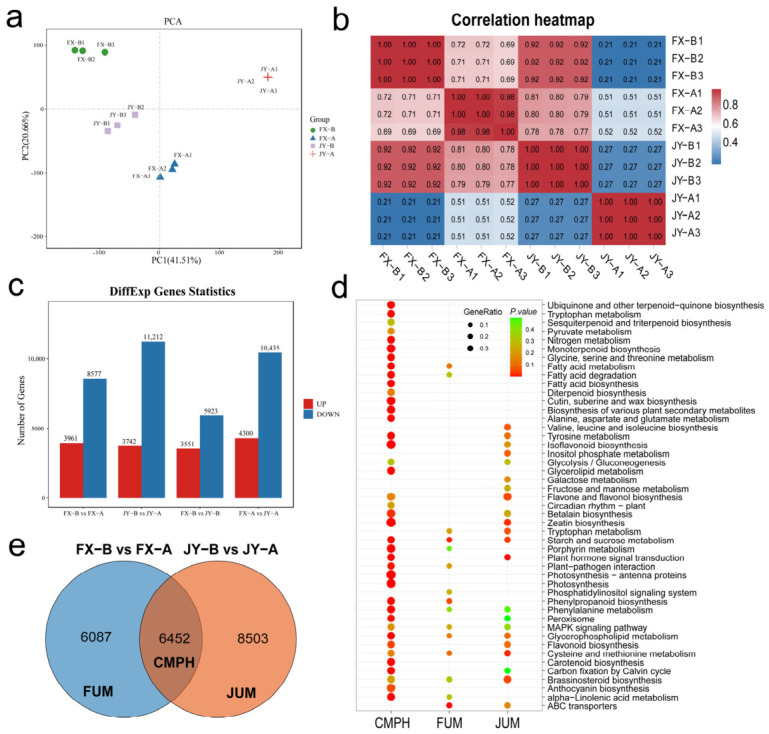
Transcriptomic analysis of leaf color transition in FX and JY. (**a**) PCA score plot based on transcriptome profiles. (**b**) Pearson correlation heatmap illustrating pairwise correlations among all samples. (**c**) Numbers of differentially expressed genes identified in each comparison. (**d**) Venn diagram showing FX-specific (FUM), JY-specific (JUM), and shared (CMPH) DEGs associated with leaf color change. (**e**) KEGG pathway enrichment bubble plot for the three DEG sets.

To characterize the transcriptional responses associated with autumn leaf coloration in sweetgum, transcriptome sequencing was conducted on leaf samples collected from the FX and JY genotypes at different seasonal stages. In total, 24 cDNA libraries were constructed and sequenced using the Illumina HiSeq 6000 platform. Each sample yielded an average of approximately 24 million high-quality reads, generating 172.54 Gbp of clean sequencing data overall. Quality assessment demonstrated that Q30 scores ranged from 92.52% to 98.47%, with a mean GC content of 45.35%, indicating high sequencing accuracy. After filtering, the clean reads were aligned to the *L. formosana* reference genome, achieving an average mapping rate of 89.87%, confirming the suitability of the dataset for downstream transcriptomic analyses. Principal component analysis (PCA) of the expression profiles (Figure 4a) showed that the first two principal components accounted for 41.51% (PC1) and 20.08% (PC2) of the total variance, respectively. Samples from different cultivars and coloration stages were clearly separated, reflecting substantial transcriptional divergence between FX and JY as well as across developmental transitions. Moreover, the tight clustering of biological replicates demonstrated strong intra-group consistency and high data reliability. Pearson correlation analysis (Figure 4b) further supported these observations, revealing very high correlations among replicates within the same group, while correlations between groups were markedly lower. The lowest correlation was observed between FX-B and JY-A samples (~0.21), underscoring pronounced differences in gene expression between cultivars at contrasting coloration stages. Differential expression analysis (Figure 4c) revealed extensive transcriptional remodeling during leaf color transition in both genotypes. In FX, a total of 12,538 differentially expressed genes (DEGs) were identified between the pre- and post-coloration stages, including 3961 upregulated and 8577 downregulated genes. In JY, 14,955 DEGs were detected over the same comparison, with 3742 genes showing increased expression and 11,212 exhibiting reduced expression. These results indicate that autumn leaf coloration is accompanied by large-scale reprogramming of gene expression in both cultivars. Venn diagram analysis (Figure 4d) further demonstrated that 6087 DEGs were unique to FX (FUM), 8503 were specific to JY (JUM), and 6452 DEGs were shared by both genotypes (CMPH). This distribution suggests the presence of a conserved transcriptional core underlying leaf color change, alongside strong cultivar-dependent regulatory components.

KEGG pathway enrichment analysis (Figure 4e) revealed striking differences in pathway utilization between the two cultivars. FX-specific DEGs (FUM) were significantly enriched in anthocyanin biosynthesis, flavone and flavonol biosynthesis, phenylpropanoid metabolism, MAPK signaling, and photosynthesis-related pathways. These enrichments suggest that FX primarily promotes flavonoid and phenylpropanoid metabolism during coloration, thereby facilitating the accumulation of flavonoids and related compounds that contribute to orange-yellow pigmentation. In contrast, JY-specific DEGs (JUM) were predominantly enriched in carotenoid biosynthesis, α-linolenic acid metabolism, steroid biosynthesis, fatty acid metabolism, and terpenoid biosynthetic pathways, indicating that red leaf coloration in JY is largely driven by enhanced carotenoid and lipid-associated metabolic processes. Shared DEGs (CMPH) were mainly involved in ubiquinone and other terpenoid-quinone biosynthesis, fatty acid metabolism, carbohydrate metabolism, and plant hormone signal transduction, reflecting fundamental metabolic and signaling pathways that support energy supply and regulatory coordination during leaf color transition in both cultivars. Collectively, these results indicate that FX and JY adopt distinct genotype-specific molecular strategies for autumn leaf coloration within a shared framework of core metabolic and hormonal regulation.

### 2.5. Integrated Transcriptomic and Metabolomic Analysis of the Anthocyanin Biosynthetic Pathway

**Figure 5:**
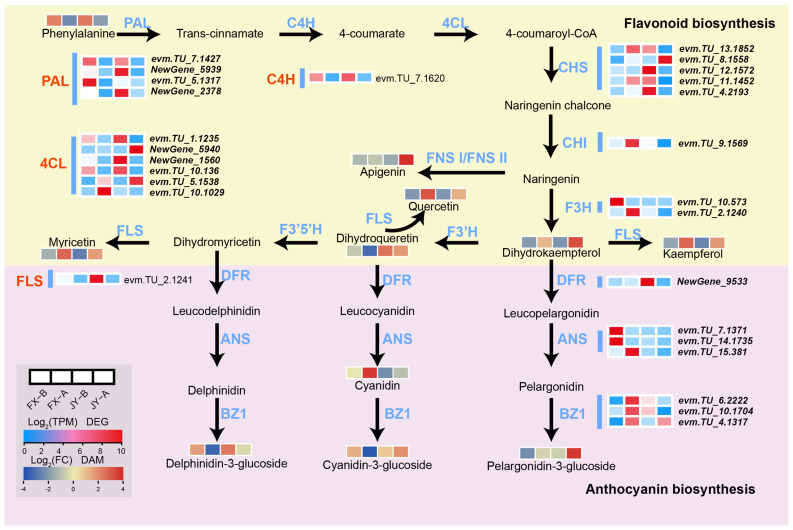
Integrated visualization of gene expression and metabolite accumulation in the flavonoid and anthocyanin biosynthetic pathways during leaf coloration in FX and JY. The schematic depicts the complete pathway from phenylalanine through phenylpropanoid and flavonoid biosynthesis to anthocyanin formation, highlighting key enzymes and intermediates. Heatmaps on the left show relative expression levels of structural genes, while those on the right illustrate corresponding changes in metabolite abundance across treatments.

During autumn leaf coloration, FX and JY exhibited markedly different regulatory patterns of anthocyanin biosynthesis. Integrated transcriptomic and metabolomic data analyses revealed substantial cultivar-dependent differences in both the expression of key structural genes and the accumulation of corresponding metabolites within the anthocyanin pathway (Figure 5). Together with the upstream entry point of the pathway, genes encoding core phenylpropanoid enzymes, including *LfPAL*, *LfC4H*, and *Lf4CL*, were activated in both genotypes. However, their expression levels and temporal profiles differed between FX and JY. Notably, *LfPAL*- and *Lf4CL*-associated genes (e.g., *evm.TU_7.1427*, *evm.TU_1.1235*) maintained relatively high expression throughout the coloration process in both cultivars, indicating sustained channeling of carbon flux into the phenylpropanoid pathway and continuous provision of precursors for downstream flavonoid and anthocyanin synthesis. In the midstream steps of the pathway, *LfCHS* and *LfCHI* genes (e.g., *evm.TU_13.1852*, *evm.TU_9.1569*) were more strongly induced in JY than in FX, thereby enhancing the conversion of chalcone intermediates into naringenin and promoting efficient formation of the flavonoid backbone. Consistently, key hydroxylase genes such as *LfF3H* and *LfF3’H* (e.g., *evm.TU_10.573*, *evm.TU_2.1240*) were also upregulated to a greater extent in JY, indicating elevated catalytic activity within the flavanone and flavanol branches and increased production of dihydrokaempferol and related intermediates. In parallel, expression of *LfFLS* declined in JY, effectively limiting carbon flux into the flavonol branch and further redirecting metabolic intermediates toward anthocyanin biosynthesis. Later downstream, rate-limiting steps of the pathway, several critical genes, including *LfDFR*, *LfANS*, and *LfBZ1* (e.g., *NewGene_9533*, *evm.TU_7.1371*, *evm.TU_6.2222*, *evm.TU_10.1704*) were markedly upregulated in JY during the coloration stage. *LfDFR* catalyzes the reduction of dihydroflavonols to leucoanthocyanidins, while *LfANS* subsequently converts these intermediates into colored anthocyanidins, and *LfBZ1* glycosylates anthocyanidins to enhance pigment stability and intensity. The coordinated high-level expression of these late biosynthetic genes provided a direct molecular basis for the dramatic transition of JY leaves from pale green to bright red.

In contrast, FX-A exhibited relatively lower expression of these downstream structural genes, suggesting that anthocyanin biosynthesis was partially constrained at the dihydroflavonol stage. As a result, a substantial proportion of metabolic flux was diverted toward the flavonol branch, leading to increased synthesis of compounds such as kaempferol and quercetin. This redistribution of carbon flux limited anthocyanin accumulation and played a role in the absence of a strong red pigmentation in FX leaves. Overall, JY-A displayed a robust and coordinated upregulation of anthocyanin biosynthesis across the entire pathway, from upstream phenylpropanoid genes to downstream *LfANS* and *LfBZ1*, forming a tightly connected transcriptional program. In contrast, FX exhibited weaker regulation at the mid- and late-stage enzymatic steps and a more diffuse allocation of metabolic resources among competing branches, ultimately resulting in insufficient anthocyanin accumulation and the development of orange-yellow foliage.

### 2.6. Identification of Transcriptional Regulatory Networks Associated with Leaf Color Variation Using WGCNA

**Figure 6:**
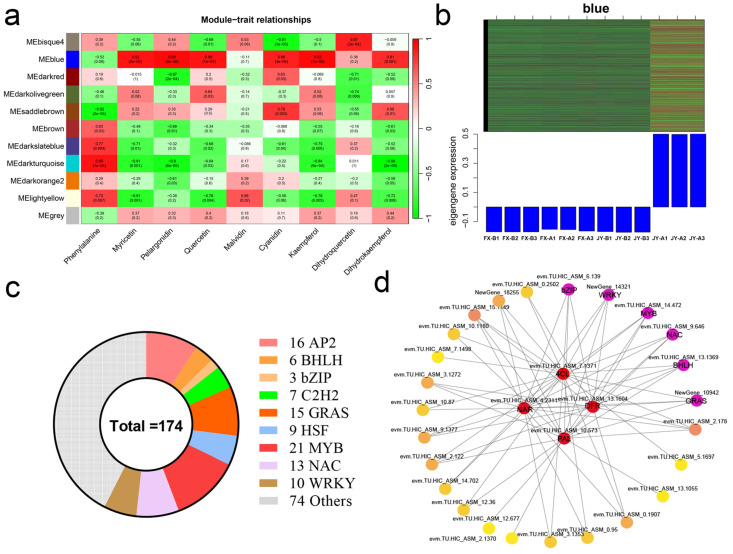
WGCNA-based gene-metabolite co-expression analysis during leaf color transition in FX and JY. (**a**) Heatmap illustrating correlations between gene co-expression modules and metabolite traits; correlation coefficients and significance levels are indicated by numerical values and color gradients (red, positive; green, negative). (**b**) Heatmap showing expression patterns of genes in the MEblue module (upper panel) and the corresponding module eigengene expression across all samples (lower panel). (**c**) Transcription factor family distribution for those represented in the MEblue module. (**d**) Co-expression-based transcriptional regulatory network linking transcription factors and key structural genes (*PAL*, *F3H*, *DFR*, and *4CL*). Node colors denote different TF families, and edge thickness reflects correlation strength.

To clarify the transcriptional mechanisms governing leaf color differentiation in the FX and JY cultivars, WGCNA was performed by integrating transcriptomic profiles with metabolomic traits. Using all expressed genes (*n* = 34,032 after low-expression filtering) and their correlations with nine representative differential metabolites (listed in Table S1), we constructed a co-expression network that resolved 12 discrete modules, each comprising between 75 and 11,957 genes. Module-trait relationship analysis revealed that several modules exhibited significant associations with metabolites involved in flavonoid and anthocyanin metabolism (Figure 6a). Among these, the blue module (MEblue) displayed the strongest positive correlations with multiple key metabolites, including phenylalanine, myricetin, pelargonidin, quercetin, malvidin, cyanidin, kaempferol, dihydroquercetin, and dihydrokaempferol. These correlations indicate that MEblue represents a central regulatory unit coordinating metabolic flux via the phenylpropanoid and flavonoid/anthocyanin biosynthetic pathways (Figure 6b).

Analysis of the module eigengene expression pattern demonstrated that MEblue was strongly activated in post-color-change JY samples, whereas its expression remained consistently low in FX samples. This cultivar-specific expression pattern closely paralleled the dramatic accumulation of anthocyanins and flavonoid derivatives observed in JY leaves following coloration. Functional annotation and transcription factor (TF) classification of genes within the MEblue module identified 174 TFs, spanning multiple families, including MYB (21 members), AP2/ERF (16), WRKY (10), HSF (9), bHLH (6), bZIP (3), and C2H2 (7) (Figure 6c). The diversity of TF families represented underscores the complexity of the transcriptional regulatory network controlling leaf color development and suggests extensive cross-talk among multiple regulatory pathways. By integrating WGCNA outputs with functional annotations, four key genes, including *PAL*, *F3H*, *DFR*, and *4CL*, were further identified as central nodes within the flavonoid and anthocyanin biosynthetic pathway. A putative transcriptional regulatory network centered on these genes was subsequently constructed. Network visualization (Figure 6d) revealed dense and highly interconnected relationships between the four structural genes and numerous TFs, with members of the MYB and WRKY families acting as prominent hubs, alongside a NAC transcription factor that also occupied a critical network position. These TFs exhibited strong co-expression correlations with the structural genes, collectively forming a tightly coordinated regulatory module. The observed associations suggest that these transcription factors likely modulate the expression of rate-limiting enzymes within the phenylpropanoid and anthocyanin pathways, thereby directing carbon flux toward anthocyanin synthesis. This coordinated regulation facilitates extensive pigment accumulation in JY leaves and underlies the progressive shift from green to purplish-red coloration. Together, these findings provide a mechanistic explanation for the contrasting autumn leaf colors of the two *L. formosana* cultivars and establish a framework for future functional studies and targeted molecular breeding efforts.

### 2.7. qRT-PCR Validation of Anthocyanin-Associated Genes in the MEblue Module

**Figure 7:**
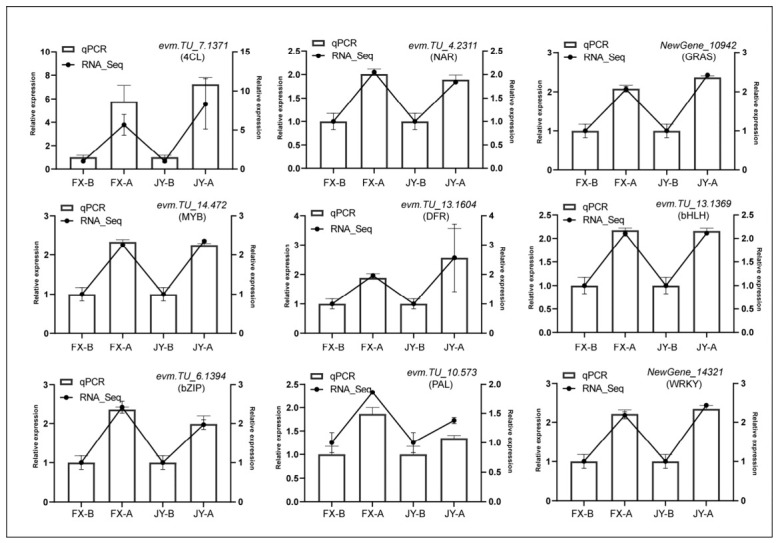
qRT-PCR validation of representative genes involved in anthocyanin biosynthesis. For each gene, bar graphs represent relative expression levels determined by qRT-PCR (left *y*-axis), while line plots indicate transcript abundance derived from RNA-Seq data (right *y*-axis). The four treatments (FX-B, FX-A, JY-B, and JY-A) are shown along the *x*-axis.

To confirm that the RNA-Seq dataset was accurate, representative structural genes and transcription factors associated with anthocyanin biosynthesis were selected from the MEblue module for qRT-PCR analysis. Although RNA-Seq and qRT-PCR employ different quantification approaches (FPKM values vs. relative expression levels), the expression patterns of all tested genes across the four sample groups (FX-B, FX-A, JY-B, and JY-A) showed strong concordance between the two methods, supporting the robustness of the transcriptomic results (Figure 7). The qRT-PCR results demonstrated that several core structural genes, including *Lf*4CL (*evm.TU_7.1371*), *LfDFR* (*evm.TU_13.1604*), and *LfPAL* (*evm.TU_10.573*), were strongly induced during the transition from green to red leaves in ‘Jinyu’ (JY-B to JY-A). In contrast, these genes exhibited only modest expression changes in wild-type *L. formosana*. These results indicate that enhanced expression of these enzyme-coding genes is a key driver of anthocyanin accumulation in JY leaves.

Consistent with the behavior of structural genes, several transcription factors, including the GRAS family member *NewGene_10942*, the MYB transcription factor *evm.TU_14.472*, the bHLH factor *evm.TU_13.1369*, the bZIP factor *evm.TU_6.1394*, and the WRKY factor *NewGene_14321*, displayed coordinated upregulation in JY-A samples. Their expression patterns closely mirrored those observed in the RNA-Seq analysis, further validating the sequencing data. The synchronized induction of these transcription factors alongside key structural genes suggests that they may function cooperatively within a regulatory network to activate anthocyanin biosynthetic genes such as 4CL, DFR, and PAL. Through this coordinated regulation, these TFs likely promote large-scale anthocyanin accumulation, thereby driving the color transition from green to red or purplish-red in ‘Jinyu’ leaves. Overall, these qRT-PCR results corroborate the reliability of the RNA-Seq analysis and reinforce the conclusion that elevated expression of both structural genes and transcription factors within the MEblue module is tightly linked to rapid anthocyanin accumulation during leaf coloration in JY. These genes are therefore inferred to play central roles in regulating anthocyanin biosynthesis and leaf color formation in *L. formosana*.

## 3. Discussion

Seasonal shifts in leaf color represent a key ecological and ornamental trait in temperate woody species, arising from coordinated changes in pigment metabolism, including chlorophyll degradation, carotenoid accumulation, and anthocyanin biosynthesis [18]. In this study, through integrated transcriptomic and metabolomic analyses of wild-type *L. formosana* (FX) and the red-leaf cultivar ‘Jinyu’ (JY), we uncover that two genotypes of the same woody species employ fundamentally distinct metabolic strategies to achieve autumn coloration–a finding that goes beyond a simple “first report” for a new species. In JY, vivid red foliage is driven by broad activation of the anthocyanin pathway, with coordinated upregulation of *LfDFR*, *LfANS*, *LfBZ1*, and significant accumulation of pelargonidin derivatives. In contrast, FX channels carbon flux toward flavonol and carotenoid metabolism, resulting in orange-yellow leaves. Moreover, WGCNA identified a co-expression module (MEblue) in which WRKY and NAC transcription factors, alongside canonical MYB and bHLH regulators, form densely interconnected hubs with key structural genes–revealing previously underappreciated regulatory components. Together with the discovery of genotype-specific anthocyanin derivatives such as pelargonidin 3-(6-p-coumaroyl)glucoside, these findings provide novel mechanistic insights and actionable targets for molecular breeding of ornamental *L. formosana*. Similar metabolic trade-offs have been reported in *Acer rubrum* and other *Acer* species [19,20], supporting the notion that selective routing of metabolic flux among competing branches is a conserved mechanism governing autumn leaf coloration in woody ornamental plants.

Metabolomic profiling revealed that, at advanced stages of leaf coloration, ‘Jinyu’ accumulated substantially higher levels of cyanidin, pelargonidin, and their glycosylated derivatives. These anthocyanins constitute the principal pigments responsible for red coloration, and their accumulation closely paralleled the transcriptional induction of key late-stage biosynthetic genes, including *LfDFR*, *LfANS*, and *LfBZ1*. This pattern is consistent with studies of fruit coloration in apple, grape, and cherry, reinforcing the pivotal role of the *LfDFR-LfANS-LfBZ1* module in regulating anthocyanin biosynthesis and accumulation [21]. In contrast, although upstream phenylpropanoid genes such as *LfPAL* and *Lf4CL* were activated in wild-type *L. formosana*, persistently elevated expression of *LfFLS* combined with comparatively weaker induction of *LfDFR* and *LfANS* redirected a substantial fraction of carbon flux toward the flavonol branch. This shift favored the accumulation of quercetin and kaempferol while constraining anthocyanin synthesis, thereby limiting red pigmentation. Competitive allocation of metabolic flux between flavonol and anthocyanin pathways has been widely documented across plant species [22], underscoring the importance of the functional balance between FLS and DFR as a key determinant of anthocyanin production efficiency. WGCNA further identified a central co-expression module (MEblue) encompassing 174 TFs, among which MYB, bHLH, WRKY, and NAC family members formed a densely interconnected network with core structural genes such as *LfPAL*, *Lf4CL*, *LfF3H*, and *LfDFR*. MYB TFs are well recognized as primary regulators of anthocyanin biosynthesis across diverse plant taxa.R2R3-MYB proteins directly activate anthocyanin structural genes by binding to their promoters, as exemplified by *PdMYB113* in poplar leaf coloration [23] and *RcMYB1* in rose [24]. In the present study, the MYB gene *evm.TU_14.472* was strongly induced during leaf coloration in ‘Jinyu’ and exhibited high co-expression with *LfDFR* and *LfANS*, suggesting it to be a major positive regulator of anthocyanin biosynthesis in *L. formosana*. Notably, the bHLH transcription factor *evm.TU_13.1369* showed a highly synchronized expression pattern with this MYB gene, implying potential formation of an MBW regulatory complex. Such complexes are widely regarded as core transcriptional modules controlling anthocyanin biosynthesis in plants [25]. The prominent representation of the WRKY transcription factor *NewGene_14321* within the MEblue module constitutes another important outcome of this study. Although WRKY proteins are classically associated with stress and defense responses, increasing evidence indicates their involvement in secondary metabolism, including flavonoid and anthocyanin biosynthesis. For example, *MdWRKY75* positively regulates anthocyanin-related genes in apple [26], *PpWRKY44* mediates light-induced anthocyanin accumulation in peach [27], and *VqWRKY56* influences flavonoid metabolism in grape [28]. These findings suggest that WRKY TFs may modulate anthocyanin biosynthesis by coordinating upstream phenylpropanoid metabolism with downstream pigment formation [29,30]. In addition, although NAC transcription factors have been less extensively studied in the context of anthocyanin regulation, emerging evidence from apple and blood orange implicates NAC proteins in pigment control, indicating that they may represent an underexplored but important regulatory node within the anthocyanin network.

The TF-structural gene network reconstructed in this study revealed strong connectivity among MYB, WRKY, and NAC TFs and key biosynthetic genes (*PAL*, *4CL*, *F3H*, and *DFR*). Such a multilayered regulatory architecture may enhance the stability and environmental responsiveness of leaf color traits in *L. formosana*. Environmental variables, including temperature, light intensity, and sugar signaling, are known to influence anthocyanin accumulation by modulating transcription factor activity. For instance, low temperatures and high irradiance can enhance TF-mediated activation of anthocyanin biosynthesis, thereby protecting photosynthetic tissues from photooxidative stress [31,32]. These observations suggest that the red coloration of ‘Jinyu’ leaves may be tightly regulated by seasonal environmental cues, whereas comparatively weaker TF activation in wild-type *L. formosana* may contribute to its limited anthocyanin accumulation. Importantly, marked metabolic and transcriptional differences between the two cultivars were evident even prior to visible color change, implying that leaf color phenotypes may be pre-established at earlier developmental stages, potentially through epigenetic regulation or cultivar-specific transcriptional programs.

In addition to differences in anthocyanin metabolism, this study showed that carotenoid and terpenoid biosynthetic pathways were preferentially enriched in wild-type *L. formosana*, whereas ‘Jinyu’ exhibited stronger activation of the anthocyanin branch. Such pathway divergence may reflect distinct adaptive strategies shaped by cultivar evolution. Carotenoids contribute to yellow pigmentation and play essential roles in photoprotection and antioxidant defense, particularly under the low-temperature and high-light conditions typical of autumn. By contrast, anthocyanins primarily function as UV and blue-green light filters and as antioxidants during stress responses. Preferential accumulation of carotenoids versus anthocyanins may therefore represent alternative adaptive solutions to environmental stress among different *L. formosana* genotypes [33]. Beyond anthocyanins, our untargeted metabolomic analysis also revealed significant variation in other phytochemical classes—including flavonols, terpenoids, and phenolic acids—that depended on both phenology and genotype (Figure 2a). This broader metabolic diversity suggests that the ornamental value of *L. formosana* is accompanied by shifts in other bioactive compounds that may have ecological or medicinal implications.

In summary, the multi-omics framework employed in this study reveals that leaf color differentiation in *L. formosana* is governed by a complex interplay among transcriptional regulation, metabolic pathway allocation, and environmental responsiveness. qRT-PCR validation confirmed the reliability of the transcriptomic data and identified key candidate genes for future functional analyses. Targeted manipulation of central transcription factors, such as MYB, bHLH, and WRKY, through genetic engineering or genome-editing approaches may enable precise modulation of leaf color traits in *L. formosana*, thereby providing both a conceptual foundation and practical strategy for molecular breeding of ornamental tree species.

## 4. Materials and Methods

### 4.1. Plant Materials, Sampling, and Treatment

Wild-type *L. formosana* (FX) and the ornamental cultivar ‘Jinyu’ (JY), which exhibits intense red foliage in autumn, were used as experimental materials in this study. All plant samples were obtained from mature trees cultivated at the Zhejiang Academy of Forestry (Hangzhou, Zhejiang Province, China). For each genotype, three healthy, uniformly growing individual trees were selected as biological replicates. Trees selected for sampling were healthy, displayed uniform growth, and showed no visible symptoms of pest infestation or disease. Fully expanded, functional leaves were collected at two distinct developmental stages corresponding to different leaf color states. Samples were harvested prior to the onset of visible color change during summer (FX-B and JY-B) and after completion of leaf coloration in autumn (FX-A and JY-A). The same three trees per genotype were sampled at both time points. For each treatment group (FX-B, FX-A, JY-B, JY-A), three independent biological replicates (one per tree) were prepared, with each replicate consisting of a pooled sample of 5–8 leaves randomly collected from the corresponding tree. Thus, a total of six trees (three for FX and three for JY) were used throughout the experiment. Immediately following harvest, leaf tissues were flash-frozen in liquid nitrogen and stored at −80 °C until subsequent metabolomic and transcriptomic analyses.

### 4.2. Metabolomic Profiling

For metabolite extraction, ~100 mg of frozen leaf tissue was homogenized using a TissueLyser II (Qiagen GmbH, Hilden, Germany) at 30 Hz for 2 min with two 5 mm stainless steel beads. The powder was extracted with 1 mL of pre-chilled extraction solvent (methanol–water, 7:3, *v*/*v*), incubated at −20 °C for 30 min, and subjected to ultrasonic extraction for an additional 30 min. After centrifugation at 13,000 rpm (15,800× *g*) for 15 min at 4 °C, the resulting supernatant was collected and passed through a 0.22 μm membrane filter prior to LC-MS/MS analysis. Metabolomic profiling was performed using a Vanquish UHPLC system (Thermo Fisher Scientific, Waltham, MA, USA) coupled to a Q Exactive HF-X Orbitrap mass spectrometer (Thermo Fisher Scientific). Chromatographic separation was carried out on an ACQUITY UPLC HSS T3 column (2.1 × 100 mm, 1.8 μm) maintained at 40 °C. The mobile phases consisted of (A) 0.1% formic acid in water (*v*/*v*) and (B) 0.1% formic acid in acetonitrile (*v*/*v*). The flow rate was set at 0.3 mL/min, and the injection volume was 2 μL. The gradient program was as follows: 0–1 min, 5% B; 1–12 min, 5% → 95% B; 12–14 min, 95% B; 14–14.1 min, 95% → 5% B; and 14.1–16 min, 5% B (re-equilibration). The mass spectrometer was operated in both positive and negative electrospray ionization (ESI) modes. The spray voltage was 3.5 kV (+) and 2.8 kV (−), the capillary temperature was 320 °C, and the sheath gas and auxiliary gas flows were 35 and 10 arbitrary units, respectively. Full MS scans were acquired at a resolution of 60,000 (m/z 100–1500), and data-dependent MS/MS fragmentation was performed at a resolution of 15,000 with a normalized collision energy of 30 eV. Raw mass spectrometry data were processed using Compound Discoverer version 3.1 (Thermo Fisher Scientific), including peak detection, retention time alignment, and metabolite annotation. Metabolite identification was achieved by integrating accurate mass measurements, MS/MS fragmentation spectra, and retention time information, and by comparison with public databases, including HMDB, KEGG, LipidMaps, and MassBank. Multivariate statistical analyses were conducted using PCA and OPLS-DA approaches. Differentially accumulated metabolites (DAMs) were identified using variable importance in projection (VIP) > 1.0 combined with Benjamini–Hochberg FDR < 0.05. The raw *p*-values are also reported for transparency, but the FDR-adjusted *p*-values were used as the final significance criterion. The detailed mass spectral data for the key metabolites, including retention time, molecular formula, measured m/z, mass error (ppm), and three major MS/MS fragment ions, are provided in Table S3. The original MS/MS spectra are also available as Figure S1.

### 4.3. Transcriptomic Analysis

Total RNA was isolated from frozen leaf samples using TRIzol (Invitrogen, Carlsbad, CA, USA). RNA concentration and purity were assessed using a NanoDrop 2000 spectrophotometer (Thermo Fisher Scientific, Waltham, MA, USA), while RNA integrity was evaluated with an Agilent 2100 Bioanalyzer (Agilent Technologies, Santa Clara, CA, USA). High-quality RNA samples were used for cDNA library construction with the NEBNext Ultra RNA Library Prep Kit for Illumina (New England Biolabs, Ipswich, MA, USA). Sequencing was conducted on the Illumina NovaSeq 6000 platform using paired-end sequencing with a 150 bp read length (PE150). Following quality filtering and removal of low-quality reads, clean reads were aligned to the Liquidambar formosana reference genome (GenBank: GCA_002263795.2) [15] using HISAT2 software (version 2.2.1), and gene expression levels were quantified using RSEM software (version 1.3.3) and normalized as FPKM values. Differentially expressed genes (DEGs) were identified based on the criteria|log_2_(Fold Change)| > 1 and an FDR < 0.05, using the Benjamini–Hochberg method. Functional enrichment analyses were conducted using GOseq for GO terms and KOBAS for KEGG pathways. Gene co-expression networks were constructed with the WGCNA package in R. DEGs were used as input to identify gene modules based on expression similarity. Module-trait and module-metabolite associations were subsequently evaluated to identify candidate genes and transcription factors potentially involved in anthocyanin biosynthesis.

### 4.4. Weighted Gene Co-Expression Network Analysis

WGCNA was carried out using the WGCNA package in R. To remove lowly expressed or noisy transcripts, only genes with FPKM ≥ 1 in at least three biological samples were retained, yielding 34,032 expressed genes for network construction. A soft-thresholding power of 6 was selected to achieve an approximate scale-free network topology. Gene modules were defined using the dynamic tree cut algorithm, with a minimum module size of 30 genes.

For each module, module eigengenes (MEs) were calculated to represent the overall expression pattern of the module. Correlation analyses were then performed between module eigengenes and nine differentially accumulated metabolites that were selected as trait variables. The selection criteria for these nine metabolites were (1) statistical significance (VIP > 1 and *p* < 0.05 in at least one pairwise comparison); (2) pathway relevance (covering upstream precursors, core flavonoid intermediates, branch-specific flavonols, and anthocyanin end-products); and (3) ability to discriminate between genotypes and coloration stages. The detailed annotation information for these nine metabolites is provided in Table S2.

### 4.5. qRT-PCR

First-strand cDNA was synthesized using the PrimeScript RT Reagent Kit (TaKaRa Bio Inc., Kusatsu, Shiga, Japan). qRT-PCR reactions were performed using TB Green Premix Ex Taq II (TaKaRa, Japan) in a total volume of 20 μL, containing 10 μL TB Green Premix, 0.8 μL of each primer (10 μM), 2 μL cDNA template, and 6.4 μL nuclease-free water. The primer sequences are provided in Table S4. The amplification protocol was 95 °C for 30 s; 40 cycles of 95 °C for 5 s and 60 °C for 30 s, followed by a melting curve analysis (95 °C for 15 s, 60 °C for 60 s, and 95 °C for 15 s). *LifEF1-α* and *LifACT* were used as internal reference genes for qRT-PCR normalization [34], and relative expression levels were computed via the 2^−ΔΔCt^ method.

### 4.6. Statistical Analyses

All experiments were performed with at least three independent biological replicates. Pairwise comparisons between two sample groups (e.g., FX-B vs. FX-A) for metabolite and qRT-PCR data were analyzed using a two-tailed Student’s *t*-test, with significance thresholds set at *p* < 0.05 (*) and *p* < 0.01 (**). Pearson correlation coefficients were calculated using R (version 4.1.2) with the Hmisc package. PCA was performed using the FactoMineR package in R. Differential expression analysis for RNA-seq data was performed using the Benjamini–Hochberg FDR method (adjusted *p* < 0.05) with the DESeq2 package. WGCNA was carried out using the WGCNA package in R. Graphical representations were generated using GraphPad Prism 8.0.

## Data Availability

The data presented in this study are available on request from the corresponding author due to the data are still undergoing in-depth analyses for ongoing follow-up studies.

## Supplementary Materials

The following supporting information can be downloaded at: https://www.mdpi.com/article/10.3390/ijms27125429/s1.

## Abbreviations

The following abbreviations are used in this manuscript:

FX	*Liquidambar formosana*
JY	*Liquidambar formosana* ‘JinYu’
