# Supplementary material for: Integrated Transcriptomic and Metabolomic Analyses Reveal the Regulatory Drivers of Anthocyanin-Mediated Leaf Color Variation in Liquidambar formosana
Source: Int J Mol Sci. 2026 Jun 16;27(12):5429. doi: 10.3390/ijms27125429 (PMC13299072; doi:10.3390/ijms27125429)
Supplement: Supplementary file 1 [file ijms-27-05429-s001.zip › Figure S1.pdf]

Figure S1. Original MS/MS spectra for the metabolites listed in Table S2.

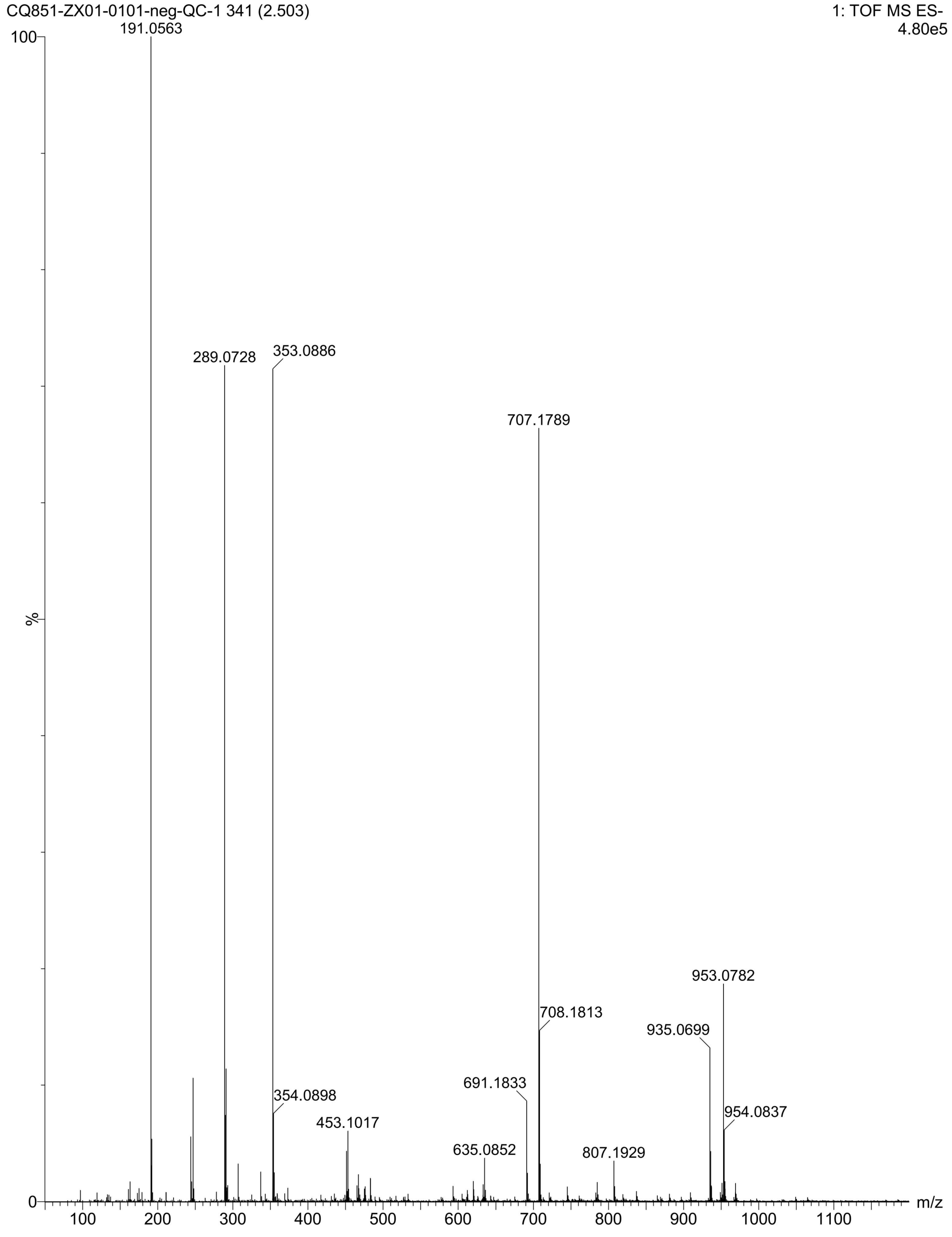

neg_2425

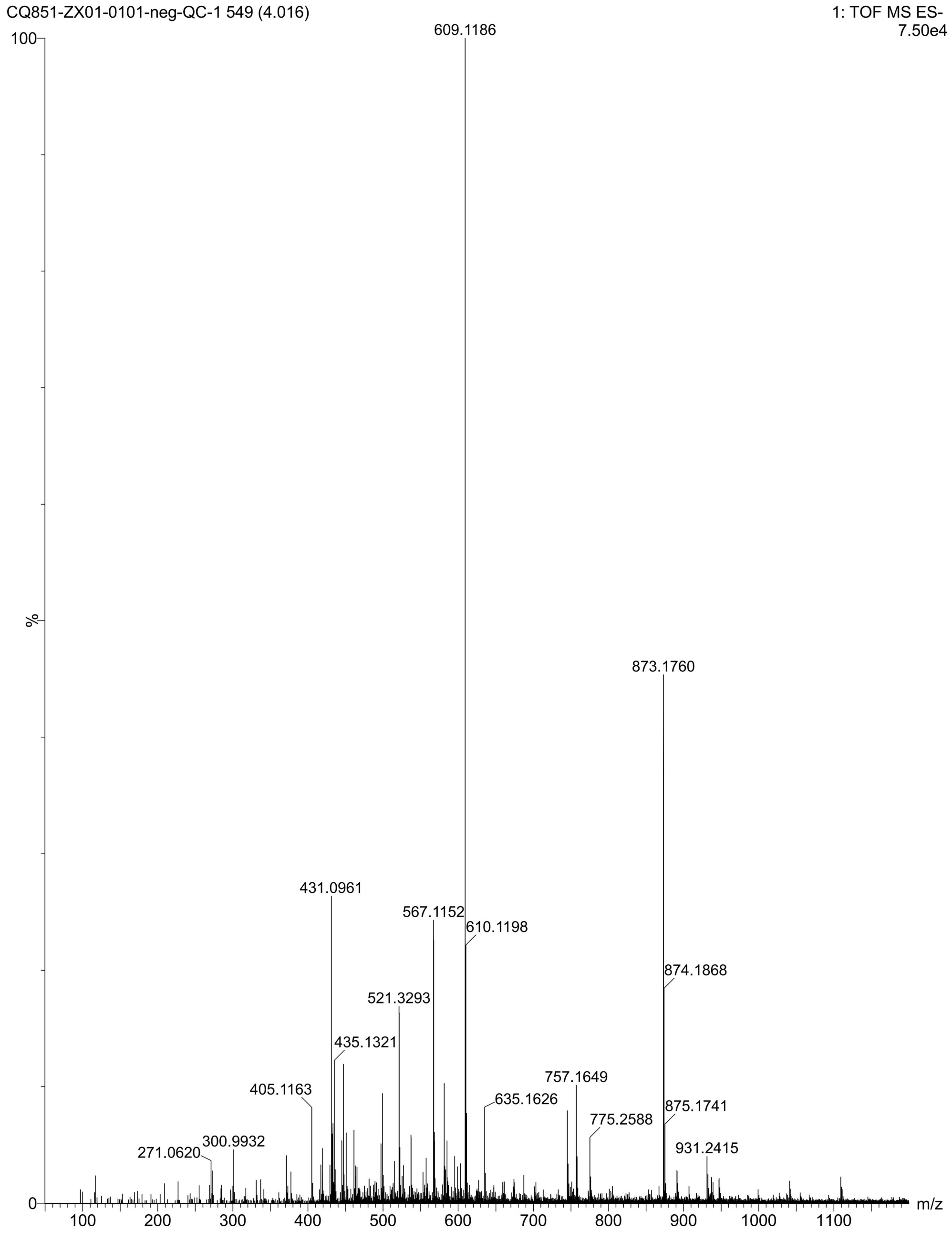
CQ851-ZX01-0101-neg-QC-1 549 (4.016)
1: TOF MS ES-
7.50e4
609.1186
873.1760
431.0961
567.1152
610.1198
874.1868
521.3293
435.1321
405.1163
757.1649
635.1626
775.2588
875.1741
271.0620
300.9932
931.2415
100
%
0
100
200
300
400
500
600
700
800
900
1000
1100
m/z

neg_4548

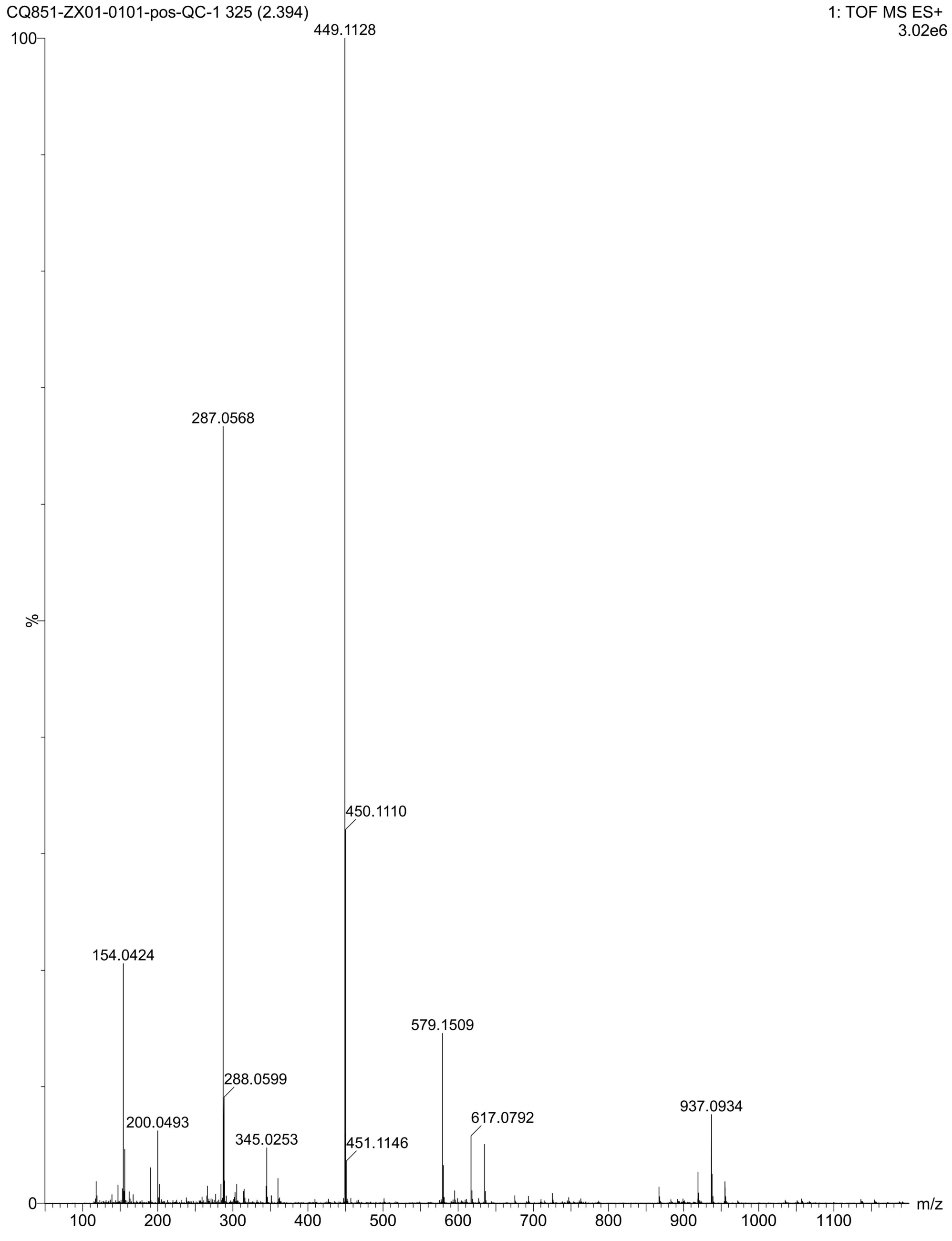
CQ851-ZX01-0101-pos-QC-1 325 (2.394)
1: TOF MS ES+
3.02e6
100
%
0
449.1128
287.0568
450.1110
154.0424
579.1509
288.0599
200.0493
617.0792
937.0934
345.0253
451.1146
m/z
100
200
300
400
500
600
700
800
900
1000
1100

pos_2197

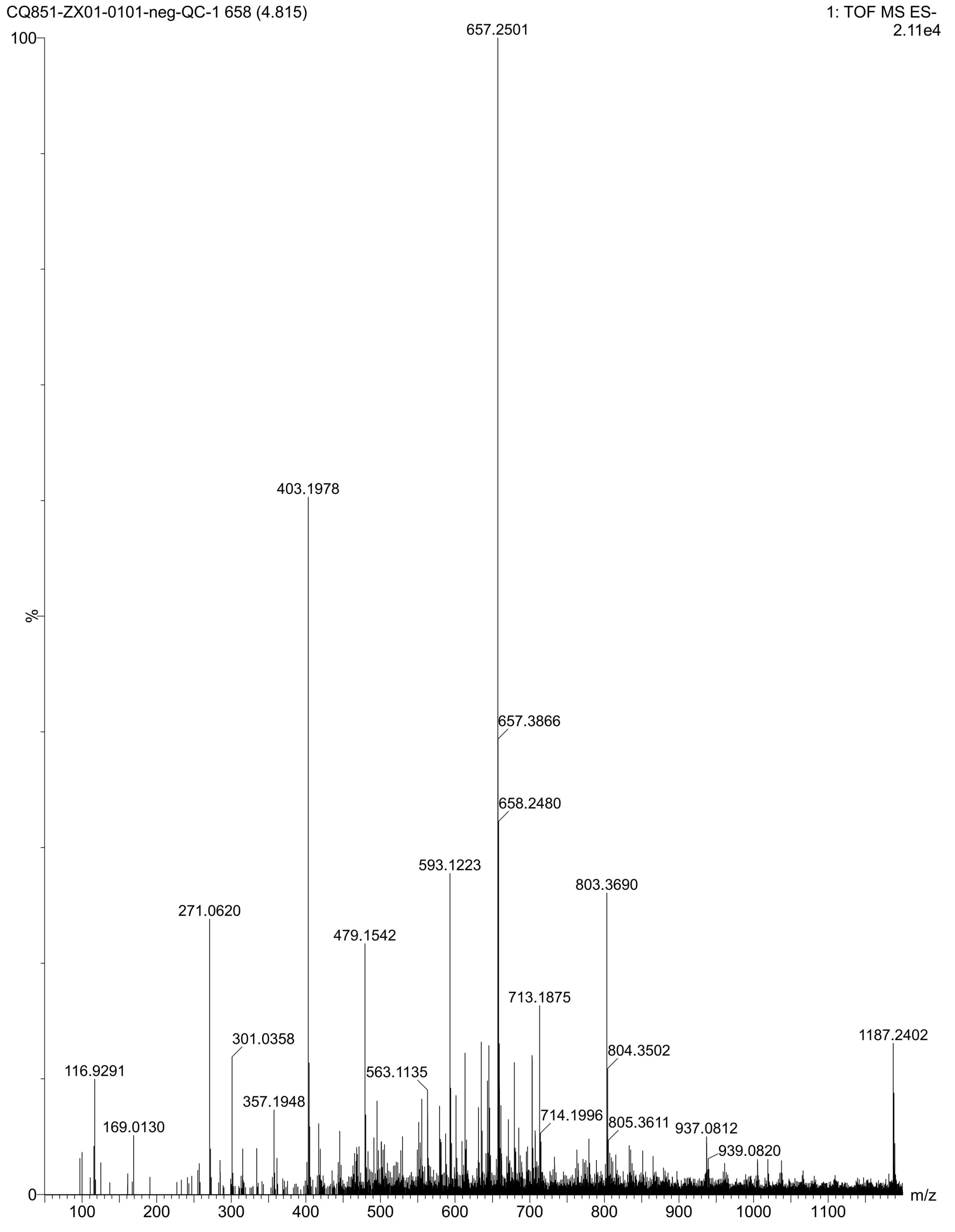
CQ851-ZX01-0101-neg-QC-1 658 (4.815)
1: TOF MS ES-
2.11e4
657.2501
403.1978
657.3866
658.2480
593.1223
803.3690
271.0620
479.1542
713.1875
301.0358
1187.2402
116.9291
804.3502
563.1135
357.1948
169.0130
714.1996
805.3611
937.0812
939.0820
100
%
0
100
200
300
400
500
600
700
800
900
1000
1100
m/z

neg_5221

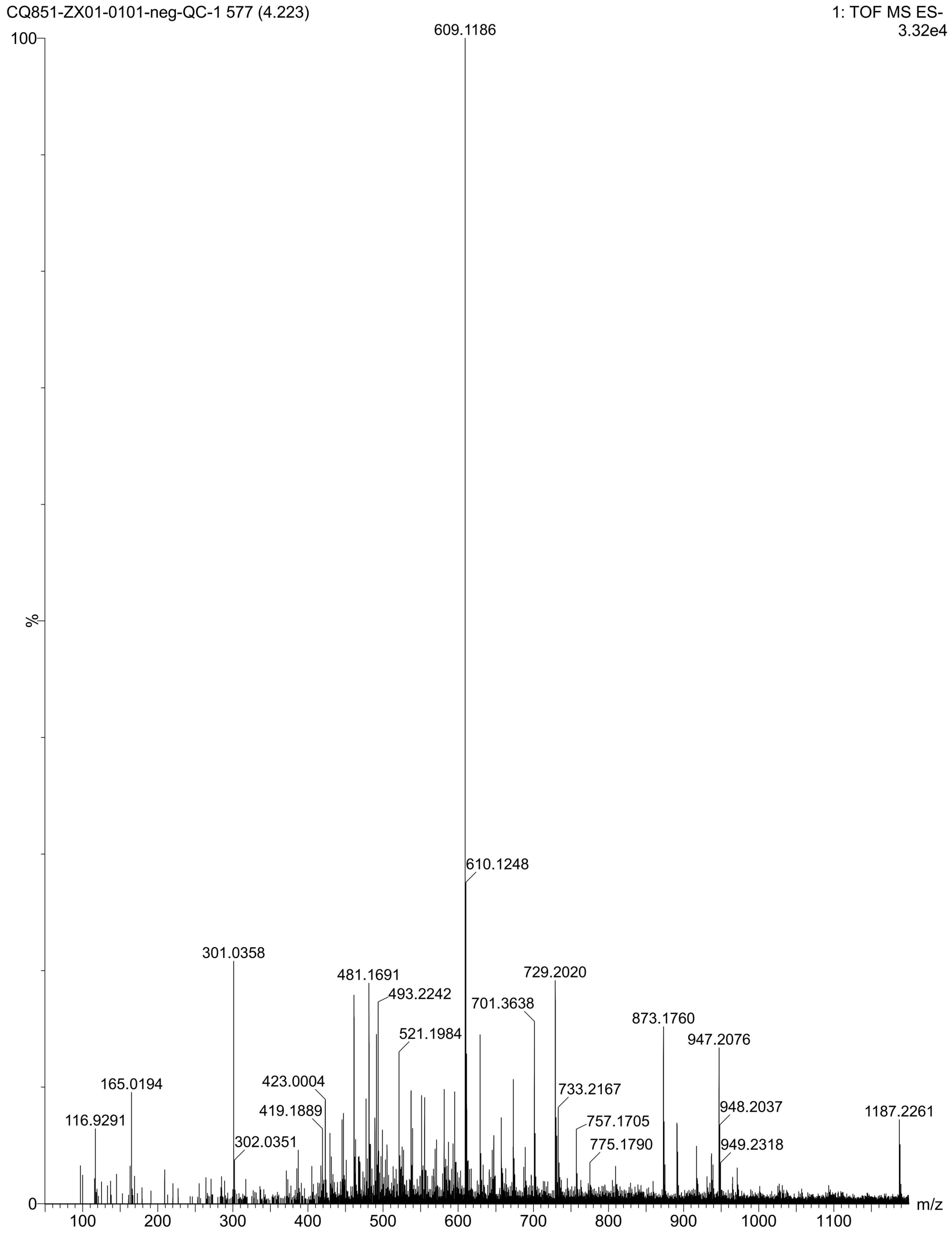
CQ851-ZX01-0101-neg-QC-1 577 (4.223)
1: TOF MS ES-
3.32e4
609.1186
610.1248
301.0358
481.1691
493.2242
729.2020
701.3638
873.1760
947.2076
521.1984
423.0004
165.0194
419.1889
733.2167
948.2037
1187.2261
116.9291
757.1705
302.0351
775.1790
949.2318
%
m/z

neg_4730

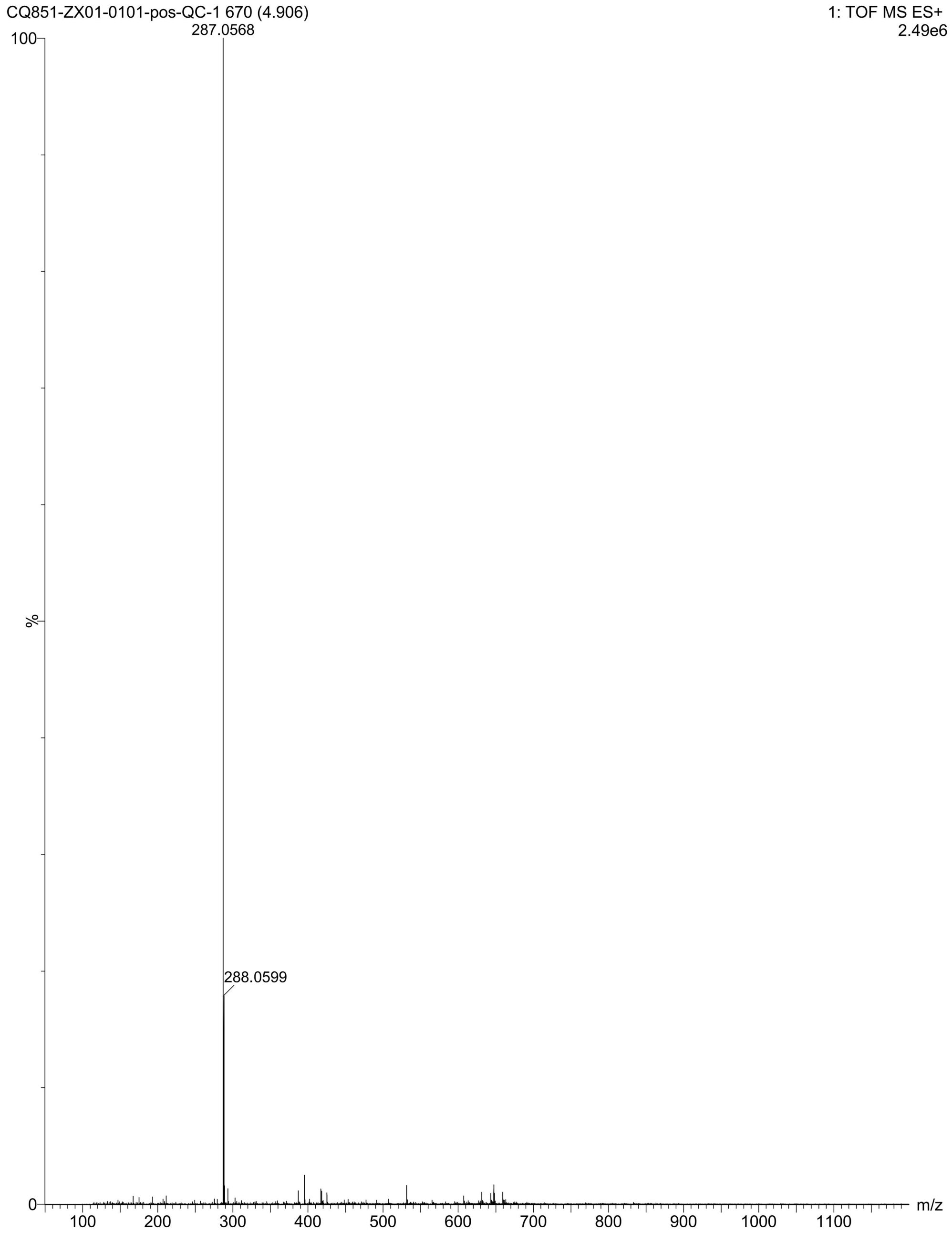
CQ851-ZX01-0101-pos-QC-1 670 (4.906)
1: TOF MS ES+
2.49e6
287.0568
288.0599
100
%
0
100
200
300
400
500
600
700
800
900
1000
1100
m/z

pos_4970

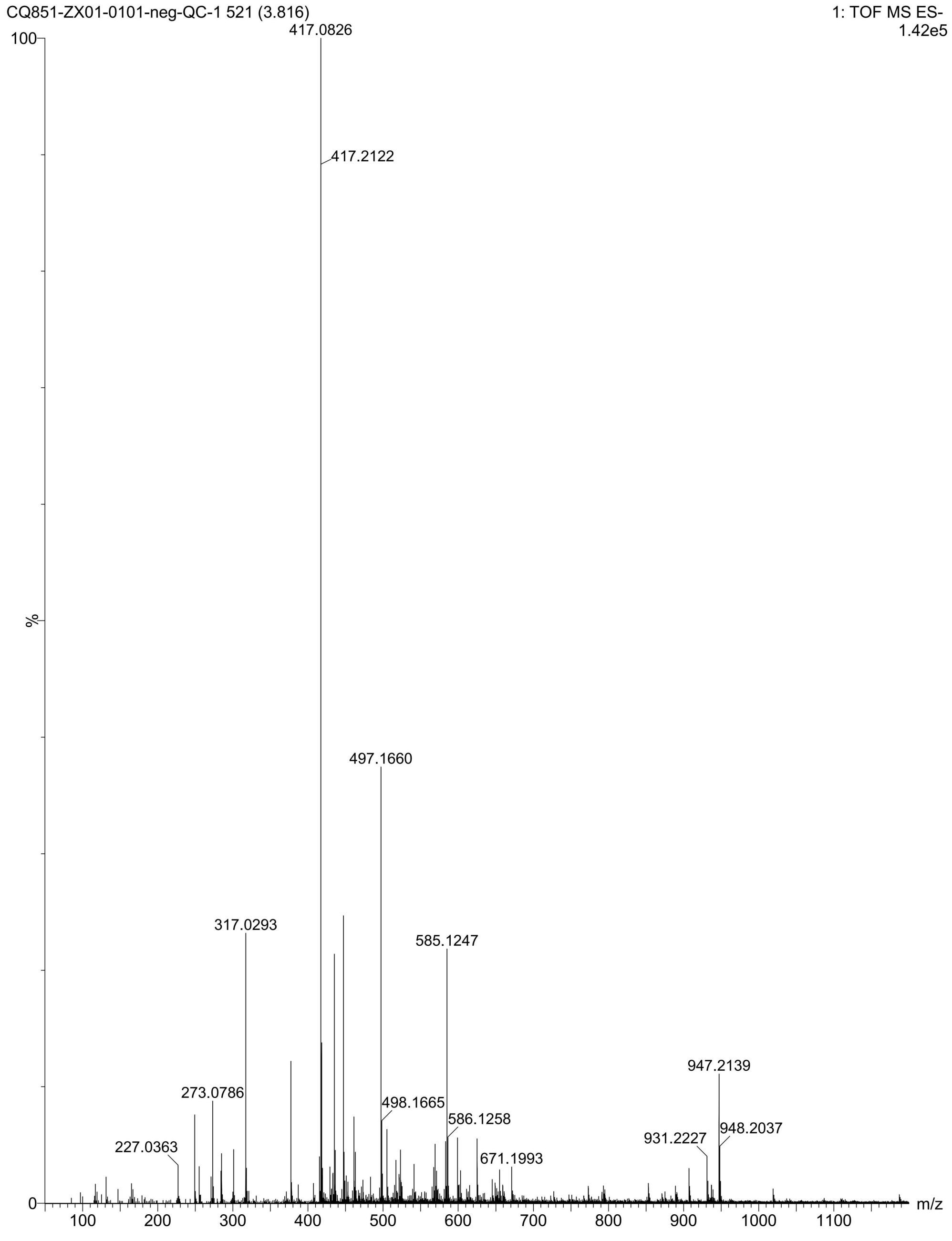
CQ851-ZX01-0101-neg-QC-1 521 (3.816)
1: TOF MS ES-
1.42e5
417.0826
417.2122
497.1660
317.0293
585.1247
947.2139
273.0786
498.1665
586.1258
931.2227
948.2037
227.0363
671.1993
100
%
0
m/z
100
200
300
400
500
600
700
800
900
1000
1100

neg_4331

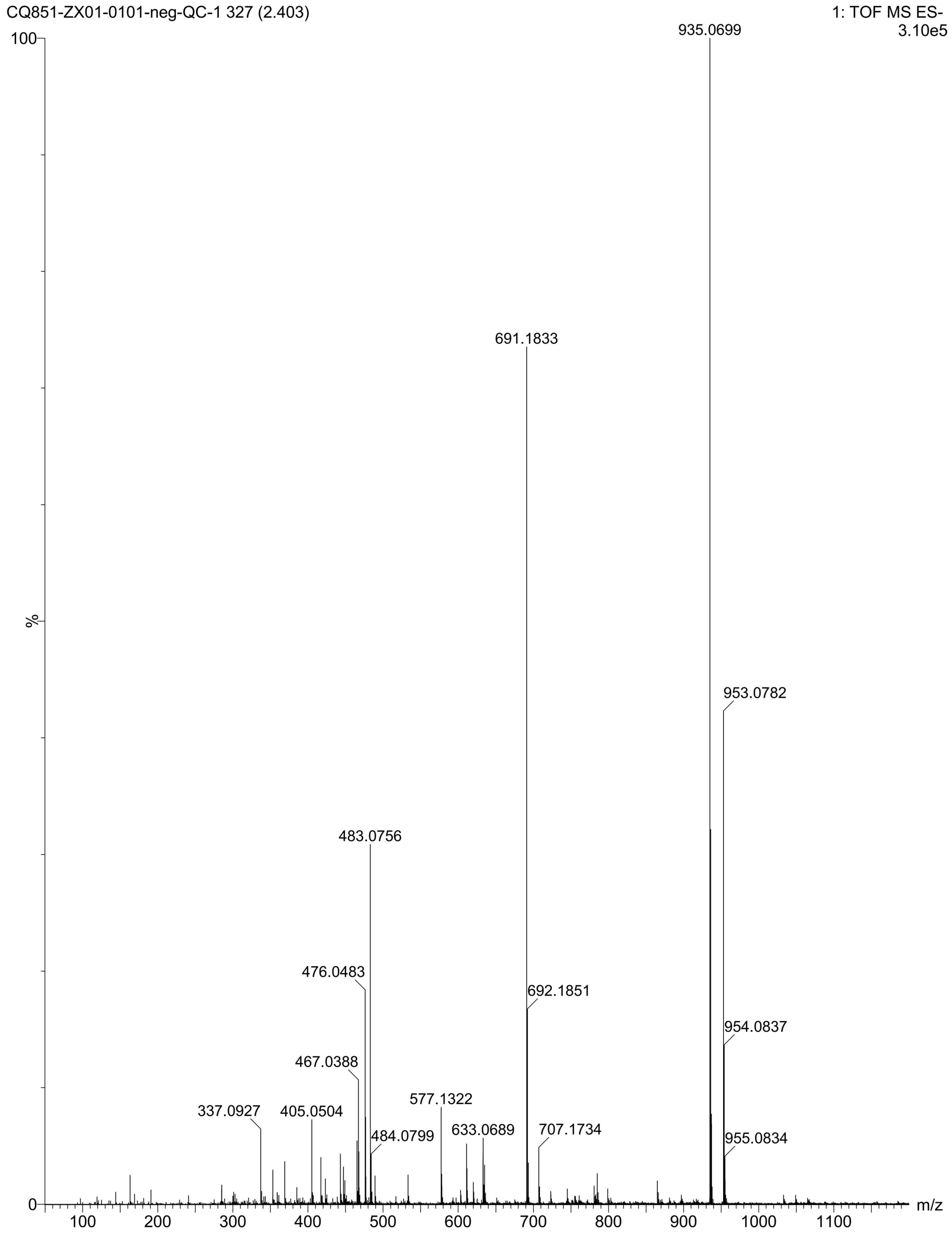

CQ851-ZX01-0101-neg-QC-1 327 (2.403)
1: TOF MS ES-
3.10e5
935.0699
691.1833
953.0782
483.0756
476.0483
692.1851
954.0837
467.0388
577.1322
337.0927
405.0504
633.0689
707.1734
484.0799
955.0834
100
%
0
100
200
300
400
500
600
700
800
900
1000
1100
m/z

neg_2256

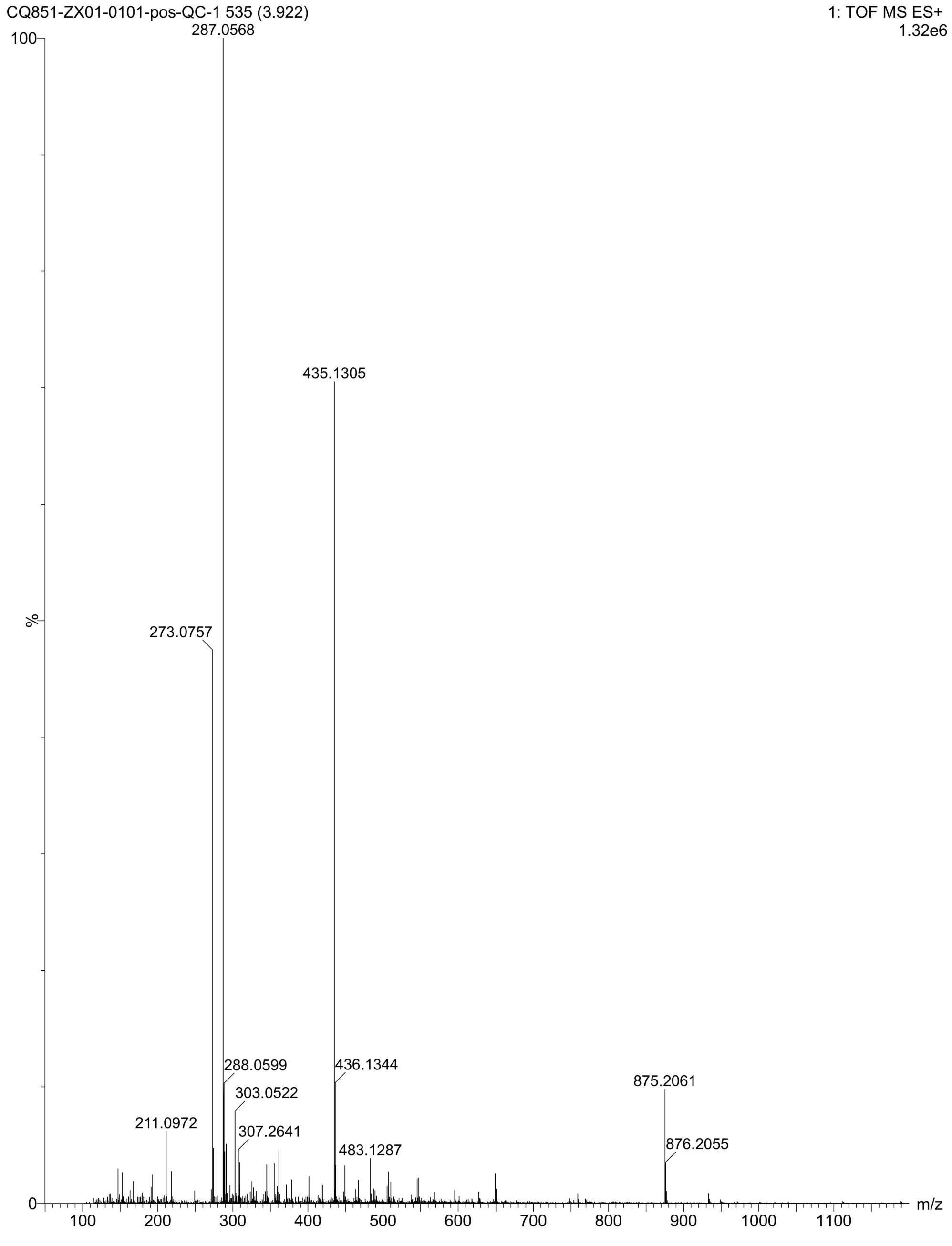
CQ851-ZX01-0101-pos-QC-1 535 (3.922)
1: TOF MS ES+
1.32e6
287.0568
435.1305
273.0757
288.0599
436.1344
303.0522
875.2061
211.0972
307.2641
483.1287
876.2055
%
100
0
100
200
300
400
500
600
700
800
900
1000
1100
m/z

pos_4102
